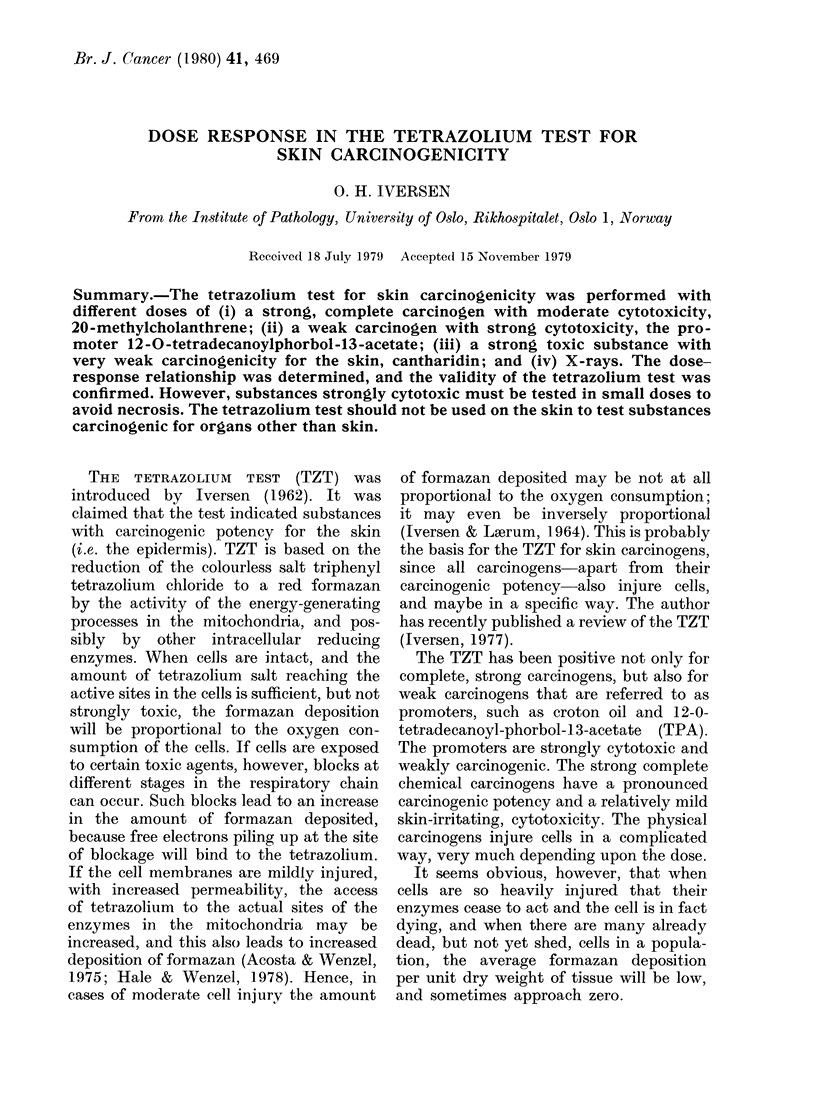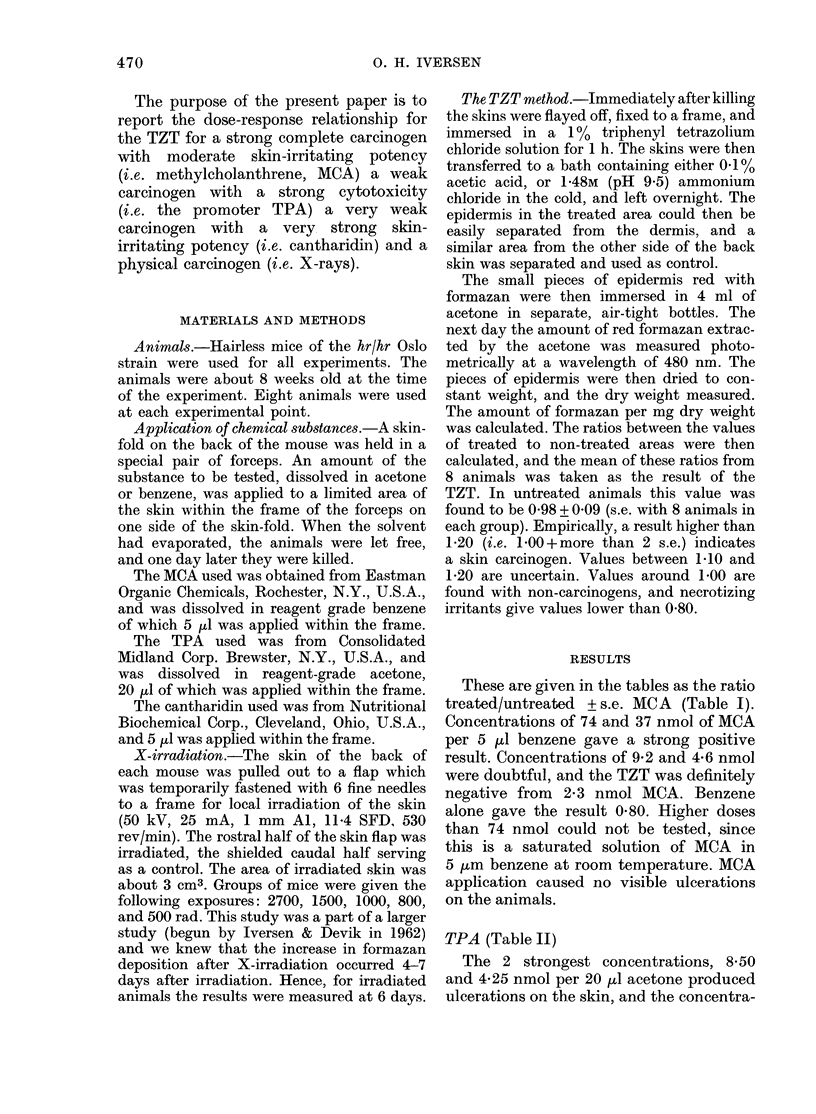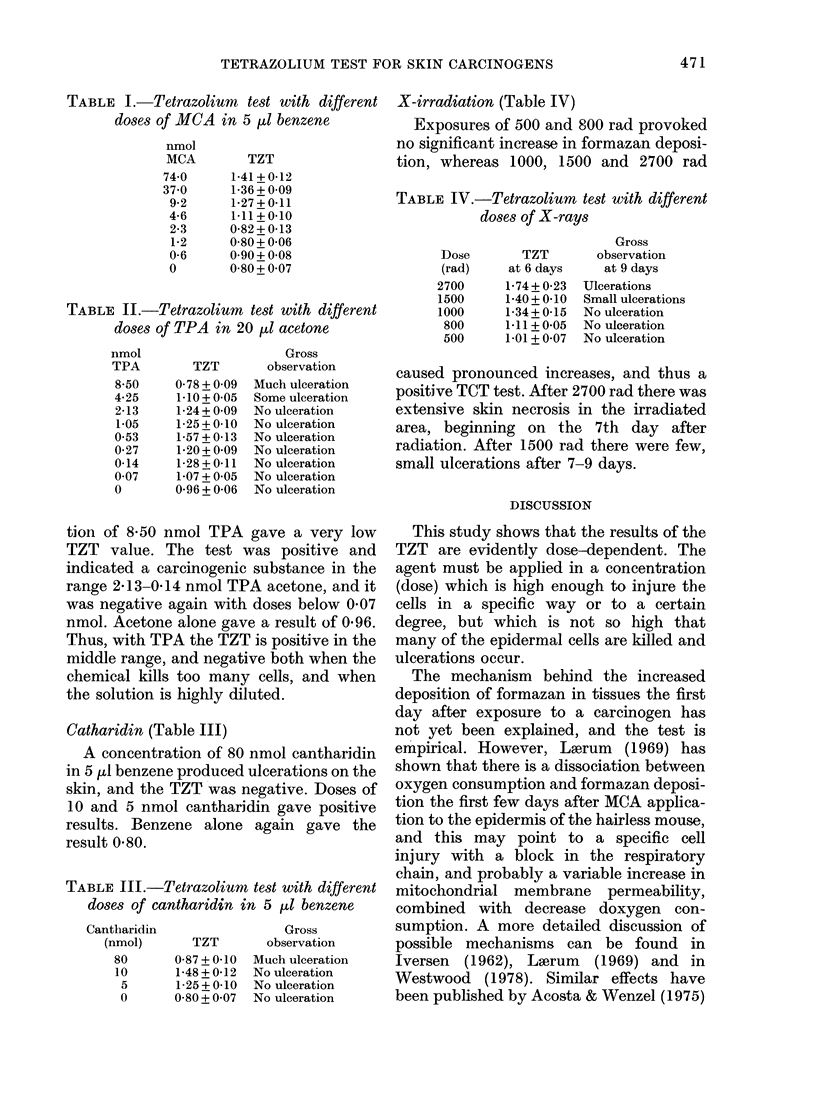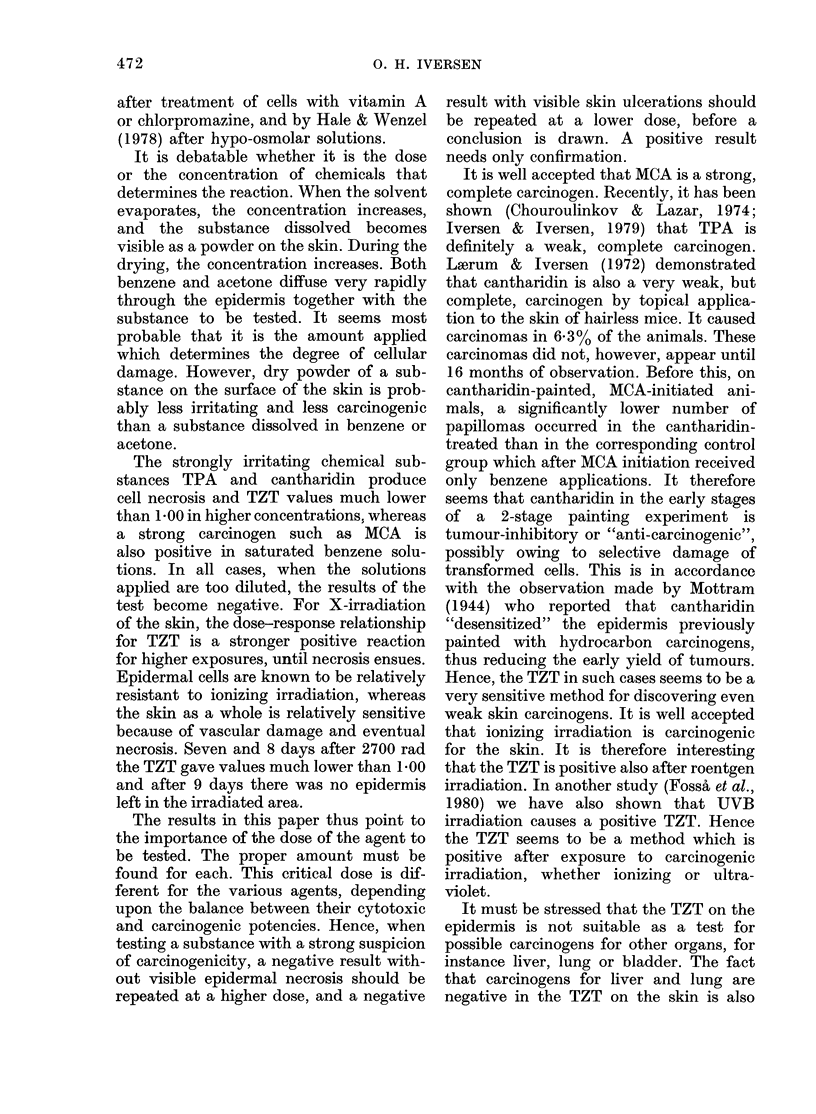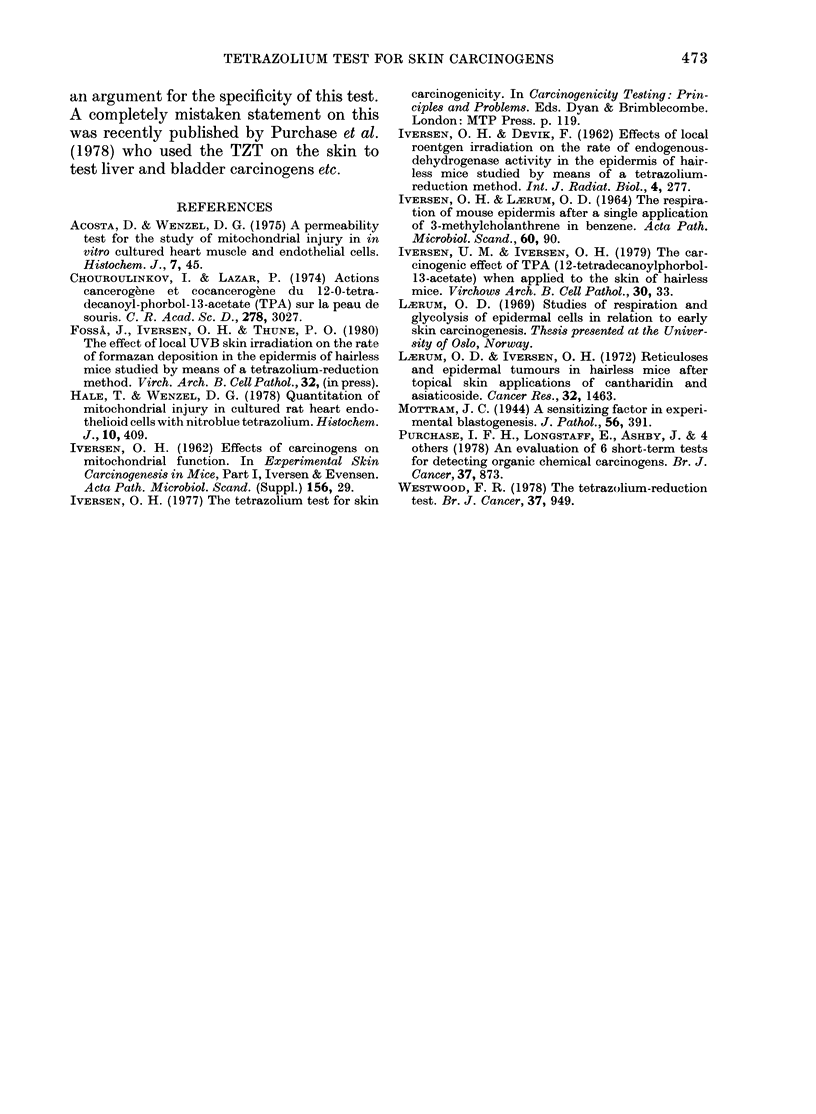# Dose response in the tetrazolium test for skin carcinogenicity.

**DOI:** 10.1038/bjc.1980.71

**Published:** 1980-03

**Authors:** O. H. Iversen

## Abstract

The tetrazolium test for skin carcinogenicity was performed with different doses of (i) a strong, complete carcinogen with moderate cytotoxicity, 20-methylcholanthrene; (ii) a weak carcinogen with strong cytotoxicity, the promoter 12-O-tetradecanoylphorbol-13-acetate; (iii) a strong toxic substance with very weak carcinogenicity for the skin, cantharidin; and (iv) X-rays. The dose-response relationship was determined, and the validity of the tetrazolium test was confirmed. However, substances strongly cytotoxic must be tested in small doses to avoid necrosis. The tetrazolium test should not be used on the skin to test substances carcinogenic for organs other than skin.


*Br. J. Cancer* (1980) **41**, 469

### DOSE RESPONSE IN THE TETRAZOLIUM TEST FOR SKIN CARCINOGENICITY

O. H. IVERSEN

*From the Institute of Pathology, University of Oslo, Rikhospitalet, Oslo 1, Norway*

Received 18 July 1979 Accepted 15 November 1979

**Summary.—The tetrazolium test for skin carcinogenicity was performed with different doses of (i) a strong, complete carcinogen with moderate cytotoxicity, 20-methylcholanthrene; (ii) a weak carcinogen with strong cytotoxicity, the promoter 12-O-tetradecanoylphorbol-13-acetate; (iii) a strong toxic substance with very weak carcinogenicity for the skin, cantharidin; and (iv) X-rays. The dose–response relationship was determined, and the validity of the tetrazolium test was confirmed. However, substances strongly cytotoxic must be tested in small doses to avoid necrosis. The tetrazolium test should not be used on the skin to test substances carcinogenic for organs other than skin.**

The TETRAZOLIUM TEST (TZT) was introduced by Iversen (1962). It was claimed that the test indicated substances with carcinogenic potency for the skin (*i.e.* the epidermis). TZT is based on the reduction of the colourless salt triphenyl tetrazolium chloride to a red formazan by the activity of the energy-generating processes in the mitochondria, and possibly by other intracellular reducing enzymes. When cells are intact, and the amount of tetrazolium salt reaching the active sites in the cells is sufficient, but not strongly toxic, the formazan deposition will be proportional to the oxygen consumption of the cells. If cells are exposed to certain toxic agents, however, blocks at different stages in the respiratory chain can occur. Such blocks lead to an increase in the amount of formazan deposited, because free electrons piling up at the site of blockage will bind to the tetrazolium. If the cell membranes are mildly injured, with increased permeability, the access of tetrazolium to the actual sites of the enzymes in the mitochondria may be increased, and this also leads to increased deposition of formazan (Acosta & Wenzel, 1975; Hale & Wenzel, 1978). Hence, in cases of moderate cell injury the amount of formazan deposited may be not at all proportional to the oxygen consumption; it may even be inversely proportional (Iversen & Lærum, 1964). This is probably the basis for the TZT for skin carcinogens, since all carcinogens—apart from their carcinogenic potency—also injure cells, and maybe in a specific way. The author has recently published a review of the TZT (Iversen, 1977).

The TZT has been positive not only for complete, strong carcinogens, but also for weak carcinogens that are referred to as promoters, such as croton oil and 12-0-tetradecanoyl-phorbol-13-acetate (TPA). The promoters are strongly cytotoxic and weakly carcinogenic. The strong complete chemical carcinogens have a pronounced carcinogenic potency and a relatively mild skin-irritating, cytotoxicity. The physical carcinogens injure cells in a complicated way, very much depending upon the dose.

It seems obvious, however, that when cells are so heavily injured that their enzymes cease to act and the cell is in fact dying, and when there are many already dead, but not yet shed, cells in a population, the average formazan deposition per unit dry weight of tissue will be low, and sometimes approach zero.

The purpose of the present paper is to report the dose-response relationship for the TZT for a strong complete carcinogen with moderate skin-irritating potency (*i.e.* methylcholanthrene, MCA) a weak carcinogen with a strong cytotoxicity (*i.e.* the promoter TPA) a very weak carcinogen with a very strong skin-irritating potency (*i.e.* cantharidin) and a physical carcinogen (*i.e.* X-rays).

### MATERIALS AND METHODS

*Animals.*—Hairless mice of the *hr/hr* Oslo strain were used for all experiments. The animals were about 8 weeks old at the time of the experiment. Eight animals were used at each experimental point.

*Application of chemical substances.*—A skin-fold on the back of the mouse was held in a special pair of forceps. An amount of the substance to be tested, dissolved in acetone or benzene, was applied to a limited area of the skin within the frame of the forceps on one side of the skin-fold. When the solvent had evaporated, the animals were let free, and one day later they were killed.

The MCA used was obtained from Eastman Organic Chemicals, Rochester, N.Y., U.S.A., and was dissolved in reagent grade benzene of which 5 μl was applied within the frame.

The TPA used was from Consolidated Midland Corp. Brewster, N.Y., U.S.A., and was dissolved in reagent-grade acetone, 20 μl of which was applied within the frame.

The cantharidin used was from Nutritional Biochemical Corp., Cleveland, Ohio, U.S.A., and 5 μl was applied within the frame.

*X-irradiation.*—The skin of the back of each mouse was pulled out to a flap which was temporarily fastened with 6 fine needles to a frame for local irradiation of the skin (50 kV, 25 mA, 1 mm Al, 11·4 SFD, 530 rev/min). The rostral half of the skin flap was irradiated, the shielded caudal half serving as a control. The area of irradiated skin was about 3 $cm^3$. Groups of mice were given the following exposures: 2700, 1500, 1000, 800, and 500 rad. This study was a part of a larger study (begun by Iversen & Devik in 1962) and we knew that the increase in formazan deposition after X-irradiation occurred 4–7 days after irradiation. Hence, for irradiated animals the results were measured at 6 days.

*The TZT method.*—Immediately after killing the skins were flayed off, fixed to a frame, and immersed in a 1% triphenyl tetrazolium chloride solution for 1 h. The skins were then transferred to a bath containing either 0·1% acetic acid, or 1·48M (pH 9·5) ammonium chloride in the cold, and left overnight. The epidermis in the treated area could then be easily separated from the dermis, and a similar area from the other side of the back skin was separated and used as control.

The small pieces of epidermis red with formazan were then immersed in 4 ml of acetone in separate, air-tight bottles. The next day the amount of red formazan extracted by the acetone was measured photometrically at a wavelength of 480 nm. The pieces of epidermis were then dried to constant weight, and the dry weight measured. The amount of formazan per mg dry weight was calculated. The ratios between the values of treated to non-treated areas were then calculated, and the mean of these ratios from 8 animals was taken as the result of the TZT. In untreated animals this value was found to be $0{\cdot}98 \pm 0{\cdot}09$ (s.e. with 8 animals in each group). Empirically, a result higher than 1·20 (*i.e.* 1·00 + more than 2 s.e.) indicates a skin carcinogen. Values between 1·10 and 1·20 are uncertain. Values around 1·00 are found with non-carcinogens, and necrotizing irritants give values lower than 0·80.

### RESULTS

These are given in the tables as the ratio treated/untreated ± s.e. MCA (Table I). Concentrations of 74 and 37 nmol of MCA per 5 μl benzene gave a strong positive result. Concentrations of 9·2 and 4·6 nmol were doubtful, and the TZT was definitely negative from 2·3 nmol MCA. Benzene alone gave the result 0·80. Higher doses than 74 nmol could not be tested, since this is a saturated solution of MCA in 5 μm benzene at room temperature. MCA application caused no visible ulcerations on the animals.

### *TPA* (Table II)

The 2 strongest concentrations, 8·50 and 4·25 nmol per 20 μl acetone produced ulcerations on the skin, and the concentra-

TABLE I.—*Tetrazolium test with different doses of MCA in 5 μl benzene*

| nmol MCA | TZT |
|---|---|
| 74·0 | 1·41 ± 0·12 |
| 37·0 | 1·36 ± 0·09 |
| 9·2 | 1·27 ± 0·11 |
| 4·6 | 1·11 ± 0·10 |
| 2·3 | 0·82 ± 0·13 |
| 1·2 | 0·80 ± 0·06 |
| 0·6 | 0·90 ± 0·08 |
| 0 | 0·80 ± 0·07 |

TABLE II.—*Tetrazolium test with different doses of TPA in 20 μl acetone*

| nmol TPA | TZT | Gross observation |
|---|---|---|
| 8·50 | 0·78 ± 0·09 | Much ulceration |
| 4·25 | 1·10 ± 0·05 | Some ulceration |
| 2·13 | 1·24 ± 0·09 | No ulceration |
| 1·05 | 1·25 ± 0·10 | No ulceration |
| 0·53 | 1·57 ± 0·13 | No ulceration |
| 0·27 | 1·20 ± 0·09 | No ulceration |
| 0·14 | 1·28 ± 0·11 | No ulceration |
| 0·07 | 1·07 ± 0·05 | No ulceration |
| 0 | 0·96 ± 0·06 | No ulceration |

tion of 8·50 nmol TPA gave a very low TZT value. The test was positive and indicated a carcinogenic substance in the range 2·13–0·14 nmol TPA acetone, and it was negative again with doses below 0·07 nmol. Acetone alone gave a result of 0·96. Thus, with TPA the TZT is positive in the middle range, and negative both when the chemical kills too many cells, and when the solution is highly diluted.

*Catharidin* (Table III)

A concentration of 80 nmol cantharidin in 5 μl benzene produced ulcerations on the skin, and the TZT was negative. Doses of 10 and 5 nmol cantharidin gave positive results. Benzene alone again gave the result 0·80.

TABLE III.—*Tetrazolium test with different doses of cantharidin in 5 μl benzene*

| Cantharidin (nmol) | TZT | Gross observation |
|---|---|---|
| 80 | 0·87 ± 0·10 | Much ulceration |
| 10 | 1·48 ± 0·12 | No ulceration |
| 5 | 1·25 ± 0·10 | No ulceration |
| 0 | 0·80 ± 0·07 | No ulceration |

*X-irradiation* (Table IV)

Exposures of 500 and 800 rad provoked no significant increase in formazan deposition, whereas 1000, 1500 and 2700 rad

TABLE IV.—*Tetrazolium test with different doses of X-rays*

| Dose (rad) | TZT at 6 days | Gross observation at 9 days |
|---|---|---|
| 2700 | 1·74 ± 0·23 | Ulcerations |
| 1500 | 1·40 ± 0·10 | Small ulcerations |
| 1000 | 1·34 ± 0·15 | No ulceration |
| 800 | 1·11 ± 0·05 | No ulceration |
| 500 | 1·01 ± 0·07 | No ulceration |

caused pronounced increases, and thus a positive TCT test. After 2700 rad there was extensive skin necrosis in the irradiated area, beginning on the 7th day after radiation. After 1500 rad there were few, small ulcerations after 7–9 days.

### DISCUSSION

This study shows that the results of the TZT are evidently dose-dependent. The agent must be applied in a concentration (dose) which is high enough to injure the cells in a specific way or to a certain degree, but which is not so high that many of the epidermal cells are killed and ulcerations occur.

The mechanism behind the increased deposition of formazan in tissues the first day after exposure to a carcinogen has not yet been explained, and the test is empirical. However, Lærum (1969) has shown that there is a dissociation between oxygen consumption and formazan deposition the first few days after MCA application to the epidermis of the hairless mouse, and this may point to a specific cell injury with a block in the respiratory chain, and probably a variable increase in mitochondrial membrane permeability, combined with decrease doxygen consumption. A more detailed discussion of possible mechanisms can be found in Iversen (1962), Lærum (1969) and in Westwood (1978). Similar effects have been published by Acosta & Wenzel (1975)

after treatment of cells with vitamin A or chlorpromazine, and by Hale & Wenzel (1978) after hypo-osmolar solutions.

It is debatable whether it is the dose or the concentration of chemicals that determines the reaction. When the solvent evaporates, the concentration increases, and the substance dissolved becomes visible as a powder on the skin. During the drying, the concentration increases. Both benzene and acetone diffuse very rapidly through the epidermis together with the substance to be tested. It seems most probable that it is the amount applied which determines the degree of cellular damage. However, dry powder of a substance on the surface of the skin is probably less irritating and less carcinogenic than a substance dissolved in benzene or acetone.

The strongly irritating chemical substances TPA and cantharidin produce cell necrosis and TZT values much lower than 1·00 in higher concentrations, whereas a strong carcinogen such as MCA is also positive in saturated benzene solutions. In all cases, when the solutions applied are too diluted, the results of the test become negative. For X-irradiation of the skin, the dose–response relationship for TZT is a stronger positive reaction for higher exposures, until necrosis ensues. Epidermal cells are known to be relatively resistant to ionizing irradiation, whereas the skin as a whole is relatively sensitive because of vascular damage and eventual necrosis. Seven and 8 days after 2700 rad the TZT gave values much lower than 1·00 and after 9 days there was no epidermis left in the irradiated area.

The results in this paper thus point to the importance of the dose of the agent to be tested. The proper amount must be found for each. This critical dose is different for the various agents, depending upon the balance between their cytotoxic and carcinogenic potencies. Hence, when testing a substance with a strong suspicion of carcinogenicity, a negative result without visible epidermal necrosis should be repeated at a higher dose, and a negative result with visible skin ulcerations should be repeated at a lower dose, before a conclusion is drawn. A positive result needs only confirmation.

It is well accepted that MCA is a strong, complete carcinogen. Recently, it has been shown (Chouroulinkov & Lazar, 1974; Iversen & Iversen, 1979) that TPA is definitely a weak, complete carcinogen. Lærum & Iversen (1972) demonstrated that cantharidin is also a very weak, but complete, carcinogen by topical application to the skin of hairless mice. It caused carcinomas in 6·3% of the animals. These carcinomas did not, however, appear until 16 months of observation. Before this, on cantharidin-painted, MCA-initiated animals, a significantly lower number of papillomas occurred in the cantharidin-treated than in the corresponding control group which after MCA initiation received only benzene applications. It therefore seems that cantharidin in the early stages of a 2-stage painting experiment is tumour-inhibitory or "anti-carcinogenic", possibly owing to selective damage of transformed cells. This is in accordance with the observation made by Mottram (1944) who reported that cantharidin "desensitized" the epidermis previously painted with hydrocarbon carcinogens, thus reducing the early yield of tumours. Hence, the TZT in such cases seems to be a very sensitive method for discovering even weak skin carcinogens. It is well accepted that ionizing irradiation is carcinogenic for the skin. It is therefore interesting that the TZT is positive also after roentgen irradiation. In another study (Fosså *et al.*, 1980) we have also shown that UVB irradiation causes a positive TZT. Hence the TZT seems to be a method which is positive after exposure to carcinogenic irradiation, whether ionizing or ultraviolet.

It must be stressed that the TZT on the epidermis is not suitable as a test for possible carcinogens for other organs, for instance liver, lung or bladder. The fact that carcinogens for liver and lung are negative in the TZT on the skin is also an argument for the specificity of this test. A completely mistaken statement on this was recently published by Purchase *et al.* (1978) who used the TZT on the skin to test liver and bladder carcinogens *etc.*

### REFERENCES

ACOSTA, D. & WENZEL, D. G. (1975) A permeability test for the study of mitochondrial injury in *in vitro* cultured heart muscle and endothelial cells. *Histochem. J.*, **7**, 45.

CHOUROULINKOV, I. & LAZAR, P. (1974) Actions cancerogène et cocancerogène du 12-0-tetradecanoyl-phorbol-13-acetate (TPA) sur la peau de souris. *C. R. Acad. Sc. D.*, **278**, 3027.

FOSSÅ, J., IVERSEN, O. H. & THUNE, P. O. (1980) The effect of local UVB skin irradiation on the rate of formazan deposition in the epidermis of hairless mice studied by means of a tetrazolium-reduction method. *Virch. Arch. B. Cell Pathol.*, **32**, (in press).

HALE, T. & WENZEL, D. G. (1978) Quantitation of mitochondrial injury in cultured rat heart endothelioid cells with nitroblue tetrazolium. *Histochem. J.*, **10**, 409.

IVERSEN, O. H. (1962) Effects of carcinogens on mitochondrial function. In *Experimental Skin Carcinogenesis in Mice*, Part I, Iversen & Evensen. *Acta Path. Microbiol. Scand.* (Suppl.) **156**, 29.

IVERSEN, O. H. (1977) The tetrazolium test for skin carcinogenicity. In *Carcinogenicity Testing: Principles and Problems.* Eds. Dyan & Brimblecombe. London: MTP Press. p. 119.

IVERSEN, O. H. & DEVIK, F. (1962) Effects of local roentgen irradiation on the rate of endogenous-dehydrogenase activity in the epidermis of hairless mice studied by means of a tetrazolium-reduction method. *Int. J. Radiat. Biol.*, **4**, 277.

IVERSEN, O. H. & LÆRUM, O. D. (1964) The respiration of mouse epidermis after a single application of 3-methylcholanthrene in benzene. *Acta Path. Microbiol. Scand.*, **60**, 90.

IVERSEN, U. M. & IVERSEN, O. H. (1979) The carcinogenic effect of TPA (12-tetradecanoylphorbol-13-acetate) when applied to the skin of hairless mice. *Virchows Arch. B. Cell Pathol.*, **30**, 33.

LÆRUM, O. D. (1969) Studies of respiration and glycolysis of epidermal cells in relation to early skin carcinogenesis. *Thesis presented at the University of Oslo, Norway.*

LÆRUM, O. D. & IVERSEN, O. H. (1972) Reticuloses and epidermal tumours in hairless mice after topical skin applications of cantharidin and asiaticoside. *Cancer Res.*, **32**, 1463.

MOTTRAM, J. C. (1944) A sensitizing factor in experimental blastogenesis. *J. Pathol.*, **56**, 391.

PURCHASE, I. F. H., LONGSTAFF, E., ASHBY, J. & 4 others (1978) An evaluation of 6 short-term tests for detecting organic chemical carcinogens. *Br. J. Cancer*, **37**, 873.

WESTWOOD, F. R. (1978) The tetrazolium-reduction test. *Br. J. Cancer*, **37**, 949.